# Modeling COVID-19 incidence with Google Trends

**DOI:** 10.3389/frma.2022.1003972

**Published:** 2022-09-15

**Authors:** Lateef Babatunde Amusa, Hossana Twinomurinzi, Chinedu Wilfred Okonkwo

**Affiliations:** Centre for Applied Data Science, College of Business and Economics, University of Johannesburg, Johannesburg, South Africa

**Keywords:** Big Data, Google Trends, ARIMA, COVID-19, infectious disease modeling

## Abstract

Infodemiologic methods could be used to enhance modeling infectious diseases. It is of interest to verify the utility of these methods using a Nigerian case study. We used Google Trends data to track COVID-19 incidences and assessed whether they could complement traditional data based solely on reported case numbers. Data on the Nigerian weekly COVID-19 cases spanning through March 1, 2020, to May 31, 2021, were matched with internet search data from Google Trends. The reported weekly incidence numbers and the GT data were split into training and testing sets. ARIMA models were fitted to describe reported weekly COVID cases using the training set. Several COVID-related search terms were theoretically and empirically assessed for initial screening. The utilized Google Trends (GT) variable was added to the ARIMA model as a regressor. Model forecasts, both with and without GTD, were compared with weekly cases in the test set over 13 weeks. Forecast accuracies were compared visually and using RMSE (root mean square error) and MAE (mean average error). Statistical significance of the difference in predictions was determined with the two-sided Diebold-Mariano test. Preliminary results of contemporaneous correlations between COVID-related search terms and weekly COVID cases reveal “loss of smell,” “loss of taste,” “fever” (in order of magnitude) as significantly associated with the official cases. Predictions of the ARIMA model using solely reported case numbers resulted in an RMSE (root mean squared error) of 411.4 and mean absolute error (MAE) of 354.9. The GT expanded model achieved better forecasting accuracy (RMSE: 388.7 and MAE = 340.1). Corrected Akaike Information Criteria also favored the GT expanded model (869.4 vs. 872.2). The difference in predictive performances was significant when using a two-sided Diebold-Mariano test (DM = 6.75, *p* < 0.001) for the 13 weeks. Google trends data enhanced the predictive ability of a traditionally based model and should be considered a suitable method to enhance infectious disease modeling.

## Introduction

The coronavirus (COVID-19) pandemic has been arguably the most critical public health challenge of the 21st century. It has had more global and rapid spread since the first confirmed cases in China in December 2019. As of February 2022, more than 434 million cases and over 5.9 million fatalities have been documented worldwide, according to the John Hopkins University (Dong et al., 2020). The COVID-19 pandemic has led to enormous social and economic harm worldwide, including job loss, severe illness, and death (Pan et al., 2020).

The coronavirus pandemic did not spare the African continent—cases have already been reported in all 54 African countries. Nigeria, in particular, reported an index case of COVID-19 on February 27, 2020, making it the first in West Africa (NCDC, 2020). Since then, the Nigerian COVID-19 cases grew steadily to more than 250,000 cases and 3,000 deaths in February 2022 (Worldometer, 2022).

The outbreak of many infectious diseases in the current digital age, including the coronavirus, has led to significant interest in using digital epidemiology and big data tools to enhance disease surveillance and modeling. Digital epidemiology, otherwise known as infodemiology, uses digital data or online sources to gain insight into disease dynamics and inform public health policies (Eysenbach, 2009; Salathé, 2018). Data used for infodemiology, which may or may not have been intended for epidemiological purposes, can be retrieved from Twitter tweets, Facebook posts, or Google search queries. Many infodemiologic studies have demonstrated the usefulness of real-time data in health assessment (Van Lent et al., 2017; Wongkoblap et al., 2017; Farhadloo et al., 2018; Lu et al., 2018; Mavragani et al., 2018b; Xu et al., 2020). Some of these studies have been used explicitly for the monitoring and forecasting of epidemics, such as Zika (Farhadloo et al., 2018), Ebola (Van Lent et al., 2017) and influenza (Lu et al., 2018).

Google Trends (GT) is the most popular Big Data surveillance tool that helps researchers analyze temporal and geographical trends in online search terms or topics (Mavragani and Ochoa, 2018b; Mavragani et al., 2018a). The Google Trends platform evaluates the popularity of top Google Search queries across multiple locations and languages. It is highly used in healthcare research for multiple health topics—A recent systematic review (Nuti et al., 2014) identified 70 peer-reviewed health-related papers studying using GT data. Several studies have used Google trends data for monitoring and forecasting disease outbreaks, including the novel coronavirus (Carneiro and Mylonakis, 2009; Mavragani and Ochoa, 2018a; Zhang et al., 2018; Mavragani and Gkillas, 2020; Rovetta and Castaldo, 2020).

A recent study (Mavragani and Gkillas, 2020) explored the predictability of COVID-19 in the US using Google Trends data. They employed a bias-corrected quantile regression model, and their results exhibited strong COVID-19 predictability. Another study (Carneiro and Mylonakis, 2009) demonstrated tracking disease activity using the Google Trends tool. Zhang et al. (2018) predicted seasonal influenza outbreaks using Google Trends and ambient temperature, and they concluded that internet search metrics combined with temperature might be utilized to forecast influenza outbreaks. Teng et al. (2017) developed an autoregressive integrated moving average model for Zika virus using search data from Google Trends. They found a strong correlation between Zika-related searches and Zika cases.

This study explored the relationship between COVID-19 cases and online interest in the virus. First, a correlation analysis between Google Trends and COVID-19 data is performed. Next, the role of Google Trends data in the predictability of COVID-19 is explored using a predictive time series model. To the best of our knowledge, this paper is the first attempt of this kind performed for Nigeria.

## Methods

### Data

We downloaded weekly incidence numbers of COVID-19 in Nigeria from the Nigeria Centre for Disease Control (https://ncdc.gov.ng). Google Trends (https://trends.google.com/trends) was used to query normalized weekly volumes of COVID-related internet searches in Nigeria. Both datasets spanned through the period March 1, 2020–May 31, 2021. We included March 1, 2020, as the initial date since the first coronavirus case in Nigeria was reported on February 27, 2020. The official COVID-19 data and Google trends internet search data used in this study are open-source and did not require permission to use.

Data retrieved from Google Trends (GT) are normalized over a defined period. Search results are proportionate to the query's time and location. The resulting numbers are on a scale of 0–100 based on a topic's proportion to all searches on all topics. A more detailed description of how Google trends data are normalized can be found elsewhere (Google Trends, 2018).

Relative search volumes (RSVs) of 15 conceptually related COVID-19 terms were assessed for online interest and the variations compared. These terms were grouped into five distinct categories (see Table 1) and compared within each group. The considered search terms have the potential to capture a broad spectrum of information related to COVID-19 (Fulk et al., 2021; Satpathy et al., 2021).

**Table 1:** Grouping of COVID-19 related GT search terms.

**Category**	**Terms**
Disease-related	“corona”, “COVID”, “COVID-19”, “coronavirus”
Symptoms	“cough”, “fever”, “loss of smell”, “loss of taste”, “sore throat”
Government instructions	“lockdown”, “quarantine”
Non-pharmaceutical interventions (NPI)	“hand wash”, “mask”, “sanitizer”, “social distancing”

The reported weekly incidence numbers and the GT data were split into training and testing sets. The training set included data from March 1, 2020, to February 28, 2021 (coinciding with the pre-vaccine arrival period), yielding 53 weeks of data. The test set included data from March 2, 2021–to May 31, 2021, yielding 13 weeks of data.

### Analysis

First, we preliminarily assessed the relationship between the GT search terms and their relationships with the official COVID-19 data. In addition to line graphs, contemporaneous correlations were assessed with Spearman rank correlation analyses, with statistical significance set at the *p* < 0.05 threshold.

Next, we modeled the COVID-19 weekly series using an autoregressive integrated moving average (ARIMA) model, which has been used to model infectious disease outbreaks (Kane et al., 2014; Johansson et al., 2016; Kandula and Shaman, 2019; Xu et al., 2020). An ARIMA (*p, d, q*) model has order *p, d, q*, corresponding to the autoregressive, differencing and moving average terms, respectively. Let *Y*_*t*_ denote the Nigerian COVID-19 cases on week *t*, the ARIMA (*p, d, q*) model can be given as


$$\begin{array}{l} D^{d}\;Y_{t}=\alpha +\sum_{k}^{p}\beta_{k}\left(D^{d}\;Y_{t-k}\right)+\sum_{k}^{q}\theta_{k}\varepsilon_{t-k}+\;\varepsilon_{t}, \end{array}$$

Where *D* is the difference operator, α is the intercept, β's and θ's are the autoregressive and moving average coefficients, respectively (Hyndman and Athanasopoulos, 2018).

An ARIMA model requires a stationary series; hence, the differencing (*d*) parameter is the number of times the series is differenced to make it stationary. The series stationarity was determined by a visual inspection of the time series plot and KPSS unit root test (Kwiatkowski et al., 1992). The AR and MA orders were identified from the autocorrelation function (ACF) and partial autocorrelation function (PACF) plots. An automatic stepwise algorithm verified the model identification by minimizing the corrected Akaike Information Criterion (AICc) (Hyndman and Khandakar, 2008). The best-fitting model was evaluated for model adequacy *via* residual diagnostic analyses. Normality of residuals was assessed with Quantile-Quantile (Q-Q) plots and Shapiro-Wilk's tests, while the ACF plot of residuals and Ljung-Box test was used for the independence of residuals.

We further develop an ARIMA model for the COVID-19 incidence by including GT for the search term(s) as a regressor. The chosen GT search terms significantly correlated (*p* < 0.05) with the official weekly cases. We performed short-term forecasts and compared them with the weekly cases in the 13-week test dataset. Prediction errors of the two models (with and without GT) were compared visually and using RMSE (root mean square error) and MAE (mean average error) values. Statistical significance of the difference in predictions was determined with the two-sided Diebold-Mariano test (Diebold and Mariano, 2002).

All statistical analyses were performed under R version 4.2.1 (R Core Team, 2020), using the forecast package version 8.4 (Hyndman and Khandakar, 2008).

## Results

**Figure 1:**
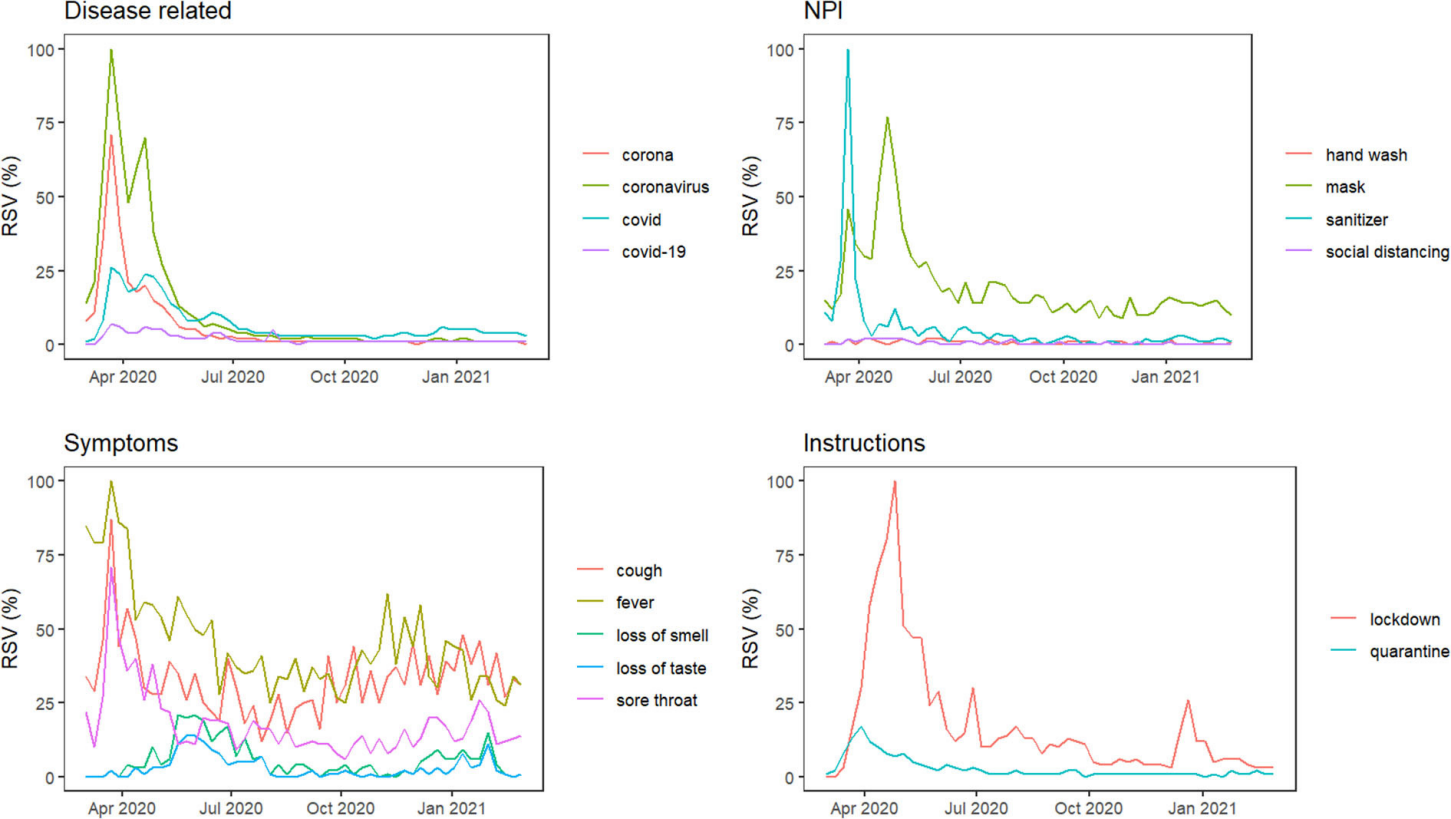
Time plot of weekly GT RSVs for some COVID-19-related search terms in Nigeria. GT RSVs, Google Trends Relative Search Volumes; NPI, Non-pharmaceutical interventions.

**Figure 2:**
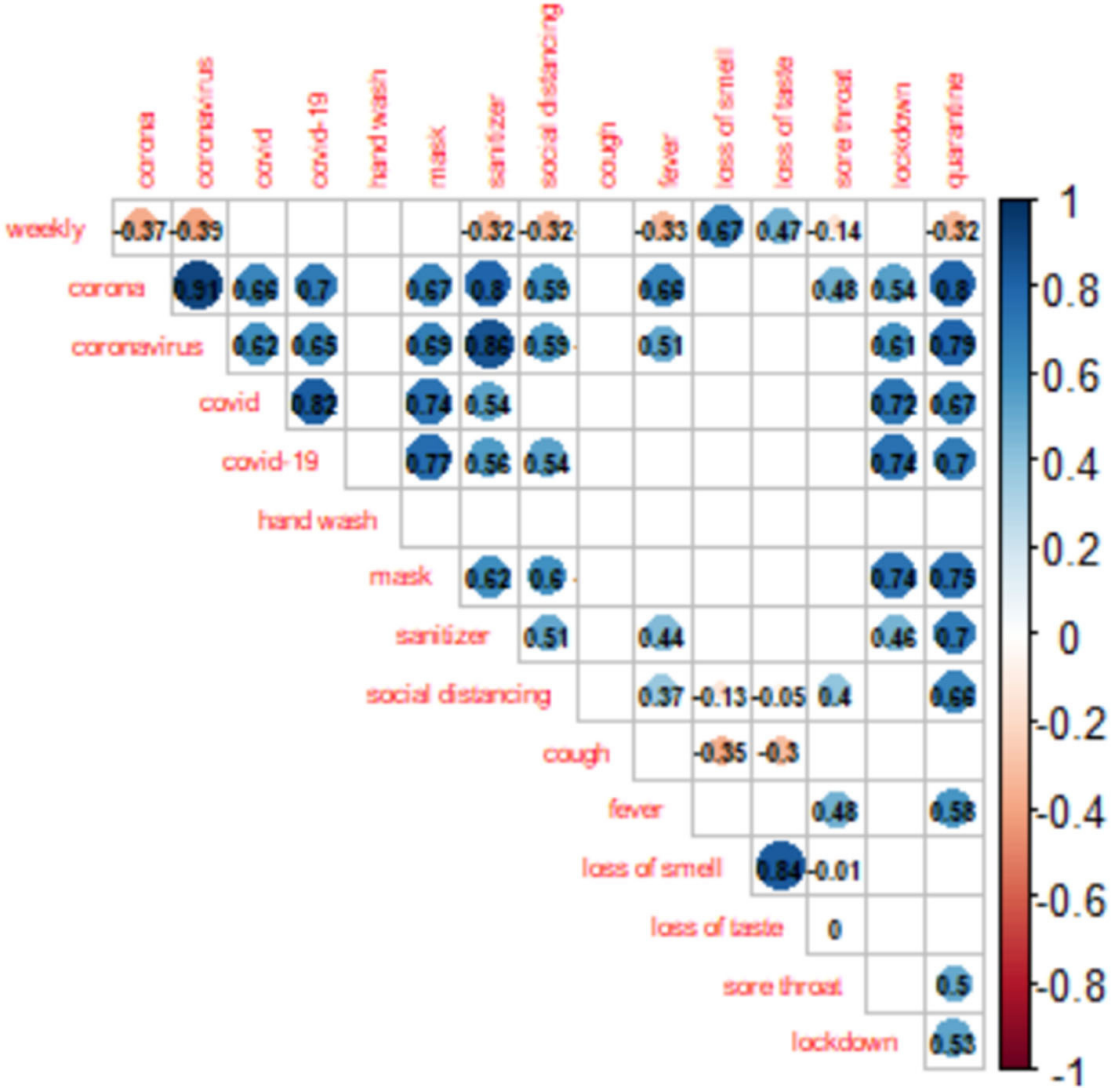
Spearman correlations among GT data and the weekly COVID-19 cases in Nigeria. The blank spaces indicate insignificant correlations (*p* < 0.05).

Figure 1 depicts Nigeria's online interest in grouped COVID-related queries from March 1, 2020, to February 28, 2021. In each group, online interest is relatively higher for “coronavirus,” “mask,” “fever,” and “lockdown.” For all the GT series, except “loss of smell” and “loss of taste”, we observed considerable peaks at the beginning of the pandemic (between the first 6 weeks) and relatively lower interests for the remainder of the series. The majority of the search terms in each group are moderately correlated (r ≥ 0.6) with each other (see Figure 2) and can be used interchangeably in further analyses.

**Figure 3:**
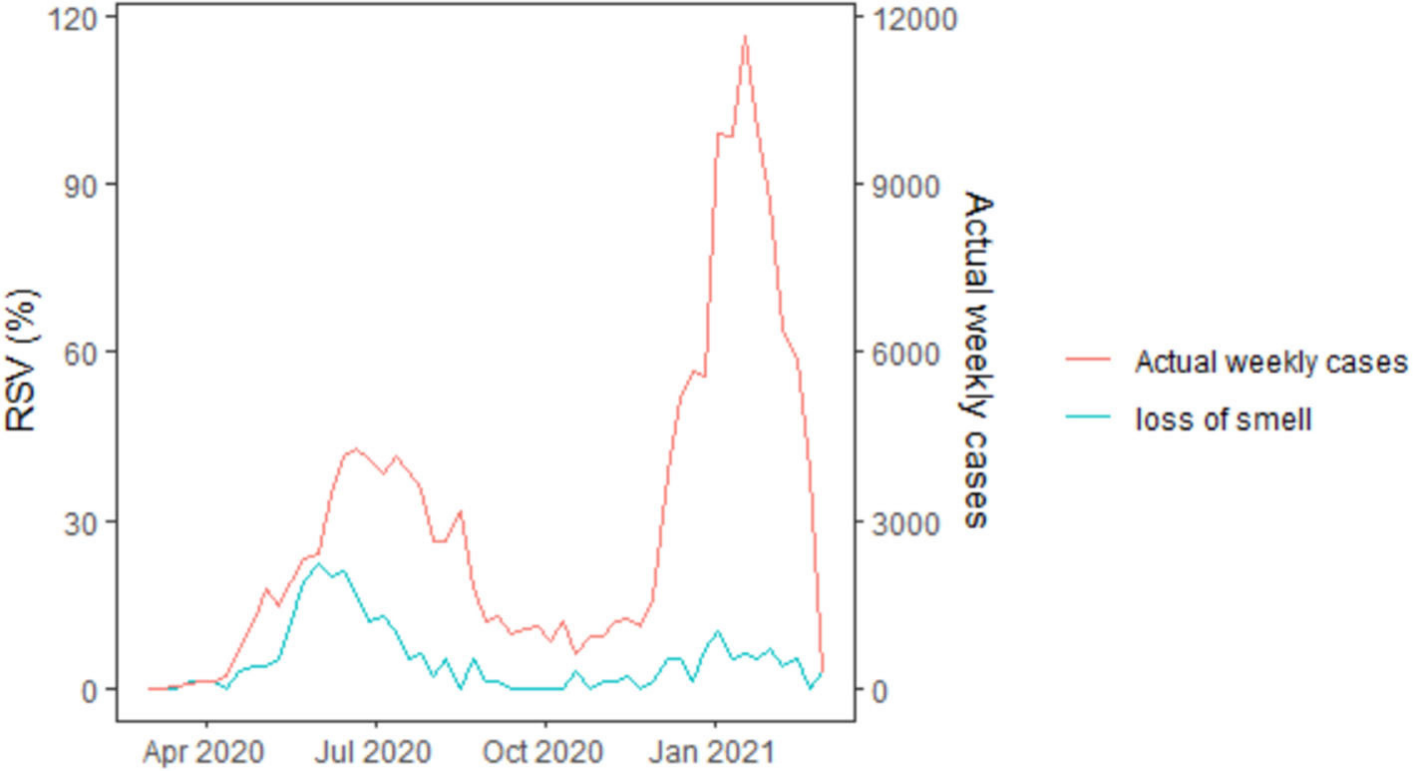
Time plot of weekly GT RSVs of “loss of smell” search term (the most strongly correlated) and the COVID-19 weekly cases in Nigeria. GT RSVs, Google Trends Relative Search Volumes.

The official weekly cases peaked in the third week of January 2021 (week 47). As shown in Figure 2, nine GT search terms showed significant contemporaneous correlations (*p* < 0.05) with the reported weekly cases, the strongest being “loss of smell” (r = 0.67, *p* < 0.001) (Figure 2). A further assessment of Figure 3 shows that the “loss of smell” GT series approximates the reported weekly cases in variation.

An initial assessment of the time plot of the COVID-19 series shows it is non-stationary. A further verification *via* the KPSS test confirms the series' non-stationarity, thus requiring differencing. The first-differenced series, however, passed the stationarity test. The KPSS results are provided in the Supplementary material.

**Figure 4:**
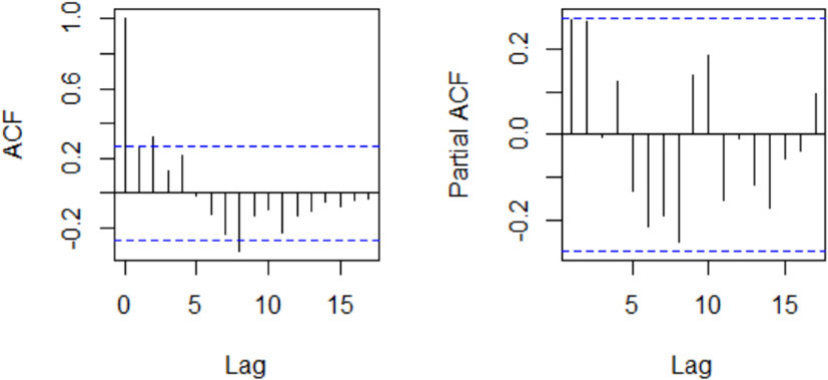
Plot of the autocorrelation and partial autocorrelation functions of the weekly COVID-19 cases.

**Figure 5:**
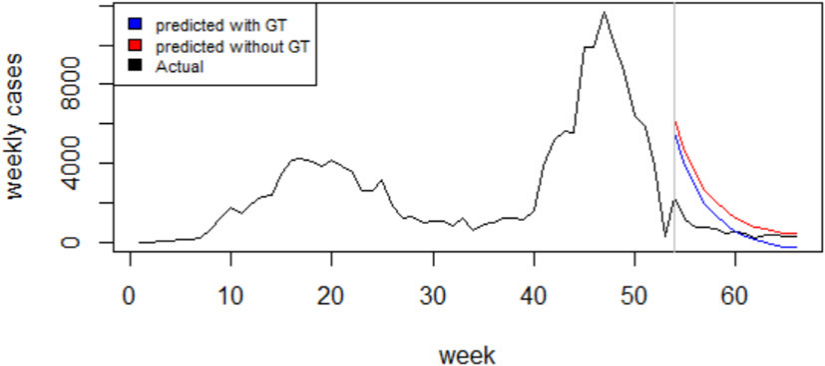
l Forecasting of the optimal ARIMA Model (red curve) compared to the Google Trends enhanced Model (blue curve) and to the actual weekly COVID-19 cases (black curve).

An examination of the ACF and PACF plots in Figure 4 suggests an ARIMA (2, 1, 0). This was confirmed by the automatic model selection procedure, which indicated ARIMA (2, 1, 0) as having the least AICc and providing the best fit to the data. A stepwise selection of the GT terms initially identified as significantly correlated with the reported weekly cases was performed, and their goodness of fit *via* AIC was assessed. Based on the results of the set of the independent variable(s) that minimized the AIC, the GT data for the search term “loss of smell” was then added as an external regressor to the ARIMA (2, 1, 0) model. Regarding model adequacy, the residuals were approximately normally distributed, and the ACF values were not significantly different from zero (Supplementary Figure S1). The Ljung-Box test result also suggests independence of the residuals (χ^2^ = 10.878; *p*-value = 0.2087). Therefore, there is sufficient evidence of the adequacy of the fitted model. The GT incorporated model performed better in terms of goodness of fit (AICc: 869.4 vs. 872.2) and forecast accuracy (Test set MAE: 340.1 vs. 354.9; Test set RMSE: 388.7 vs. 411.4). Model comparison results are presented in Table 2. Figure 5 compares the reported weekly cases with the forecast values from the two different models. Though the model predictions are similar in pattern, they are quite different in values. The two-sided Diebold Mariano test provided evidence of a significant difference (DM = 6.75, *p* < 0.001) in the predictive performance of the two models.

**Table 2:** Comparative performance assessment of the model without GT and the GT-enhanced model.

	**Model without GT**		**Model with GT**	
AICc	872.2		869.4	
Training set RMSE	253.7		231.8	
Test set RMSE	411.4		388.7	
Training set MAE	190.8		176.6	
Test set MAE	354.9		340.1	
**Parameters**	**Coefficient (S.E)**	***P*** **-value**	**Coefficient (S.E)**	***P*** **-value**
AR1	0.255 (0.139)	0.066	0.249 (0.139)	0.072
AR2	0.361 (0.143)	0.012	0.378 (0.144)	0.009
GT	NA		9.065 (12.034)	0.451

AR, Autoregressive; SE, Standard error; MAE, Mean absolute error; RMSE, Root mean squared error; AICc, corrected Akaike information criterion.

## Discussion

We examined the utility of online search data, *via* Google Trends (GT), for improving the forecasting accuracy of official COVID-19 cases, focusing on Nigeria. Little to no research evaluate the predictive performance of models based upon GT data for African contexts. Internet penetration in Africa, compared with other continents, remains low, and such a study, therefore, represents a significant contribution (Fulk et al., 2021).

Our preliminary results of the Spearman rank correlation analysis found that many (9 out of 15) search terms had significant contemporaneous correlations with the COVID-19 case numbers. Two previous studies (Mavragani and Gkillas, 2020; Satpathy et al., 2021) agree with the identified significant contemporaneous correlations. The inclusion of GT data significantly improved the predictive accuracy of the fitted ARIMA model.

Google Trends has proven extremely useful in researching widespread interest in health-related topics (Nuti et al., 2014), specifically infectious diseases (Carneiro and Mylonakis, 2009; Zhang et al., 2018; Mavragani and Gkillas, 2020; Rovetta and Castaldo, 2020). Notably, most of these studies (Nuti et al., 2014) only examined correlations between GT data and official incidence numbers. However, this study is of particular interest, given the relative paucity of GT studies in modeling or prediction. Only two studies (Ayyoubzadeh et al., 2020; Mavragani and Gkillas, 2020) modeled COVID-19 incidence from a further dissection of the GT studies that performed disease modeling. Mavragani and Gkillas (2020) employed a bias-corrected quantile regression model to explore the predictability of COVID-19 in the US using Google Trends data. Ayyoubzadeh et al. (2020) used Linear regression and long short-term memory (LSTM) models to predict COVID-19 incidence in Iran.

Our preliminary analyses revealed that symptoms search terms are more reliable correlates of COVID-19. We found loss of smell and taste as the most predictive symptom of COVID-19 infection. This agrees with the findings of Cherry et al. (2020). They demonstrated a clear association between COVID-19 cases and GT search terms relating to the loss of smell and taste on a regional, national, and international basis.

Here, we utilized ARIMA modeling due to its reputation as one of the most reliable time series analysis methods for infectious diseases (Allard, 1998; Song et al., 2016). Further, the ARIMA model is relatively straightforward and can be utilized by applied researchers with minimal modeling expertise. Other GT studies similar to ours used the seasonal version of the ARIMA model (SARIMA), which is ideal for seasonal conditions such as pertussis (Nann et al., 2021), tick-borne encephalitis (Sulyok et al., 2020), dengue fever (Wongkoon et al., 2012), malaria (Midekisa et al., 2012), and hepatitis E (Ren et al., 2013).

Compared to other Big Data platforms, the strength of Google Trends data lies in the ease of access. This study is not without limitations. The major limitation of this study is that there is no provision for the exact methodology for data generation, and the study population responsible for the searches cannot be determined. Therefore, we cannot control for possible confounders, including environmental and demographic factors that may impact search activity and COVID-19 incidence. More accurate and informative models could be developed if at least the absolute search frequency is available. Furthermore, related media activities can substantially influence search volumes, thereby lacking reliability with epidemiologic occurrence.

Selection bias is possible in obtaining RSV data since we used some selected COVID-19-related keywords, which may have been incomplete. Further research could aim to identify the most relevant set of keywords. These limitations point to the need to interpret this study's findings with caution. Despite the known limitations of online search data, its usage for informing public health and policy in general and monitoring outbreaks and epidemics, particularly, has received wide attention.

## Conclusion

It is important to note that while the easy-to-obtain Google search data is a more dynamic and available source than traditional data sources, we have used the results from GT data to supplement the traditional data rather than replacing it. We tested whether the inclusion of GT data improves the routine epidemiologic methods. In conclusion, GT data correlate with the reported incidence of COVID-19 in Nigeria, significantly improving forecasting accuracy in the models based on traditional data. Efficient use of online search data could anticipate future rises in disease incidence and possibly more timely allocation of healthcare resources. Future studies can replicate this study with other data sets and forecasting methodologies. Modeling with different algorithms, analyzing data from other regions and countries, or even spatial analyses are potential future perspectives.

## Data availability statement

The datasets presented in this study can be found in online repositories. The names of the repository/repositories and accession number(s) can be found below: https://github.com/amusasuxes/gtrends/blob/main/gtrends.

## Author contributions

LA designed the study, analyzed the data, and wrote the manuscript. CO assisted with some relevant literature. HT critically reviewed the manuscript and gave constructive comments, which improved the manuscript. All authors have read and approved the manuscript.

## Conflict of interest

The authors declare that the research was conducted in the absence of any commercial or financial relationships that could be construed as a potential conflict of interest.

## Publisher's note

All claims expressed in this article are solely those of the authors and do not necessarily represent those of their affiliated organizations, or those of the publisher, the editors and the reviewers. Any product that may be evaluated in this article, or claim that may be made by its manufacturer, is not guaranteed or endorsed by the publisher.

## Abbreviations

GT: Google Trends
COVID-19: Coronavirus 2019
ARIMA: Autoregressive integrated moving average
RSV: Relative search volume.

## References

[B1] Allard R. (1998). Use of time-series analysis in infectious disease surveillance. Bull. World Health Organ. 76, 327. 9803583 PMC2305771

[B2] Ayyoubzadeh S. M. Ayyoubzadeh S. M. Zahedi H. Ahmadi M. Kalhori S. R. N. (2020). Predicting COVID-19 incidence through analysis of google trends data in iran: data mining and deep learning pilot study. JMIR Public Health Surveill. 6, e18828. 10.2196/18828 32234709 PMC7159058

[B3] Carneiro H. A. Mylonakis E. (2009). Google trends: a web-based tool for real-time surveillance of disease outbreaks. Clin. Infect. Dis. 49, 1557–1564. 10.1086/630200 19845471

[B4] Cherry G. Rocke J. Chu M. Liu J. Lechner M. Lund V. J.. (2020). Loss of smell and taste: a new marker of COVID-19? Tracking reduced sense of smell during the coronavirus pandemic using search trends. Expert Rev. Anti. Infect. Ther. 18, 1165–1170. 10.1080/14787210.2020.1792289 32673122 PMC7441792

[B5] Diebold F. X. Mariano R. S. (2002). Comparing predictive accuracy. J. Bus. Econ. Stat. 20, 134–144. 10.1198/073500102753410444

[B6] Dong E. Du H. Gardner L. (2020). An interactive web-based dashboard to track COVID-19 in real time. Lancet Infect. Dis. 20, 533–534. 10.1016/S1473-3099(20)30120-1 32087114 PMC7159018

[B7] Eysenbach G. (2009). Infodemiology and infoveillance: framework for an emerging set of public health informatics methods to analyze search, communication and publication behavior on the Internet. J. Med. Internet Res. 11, e1157. 10.2196/jmir.1157 19329408 PMC2762766

[B8] Farhadloo M. Winneg K. Chan M.-P. S. Jamieson K. H. Albarracin D. (2018). Associations of topics of discussion on Twitter with survey measures of attitudes, knowledge, and behaviors related to Zika: probabilistic study in the United States. JMIR Public Health Surveill. 4, e8186. 10.2196/publichealth.8186 29426815 PMC5889815

[B9] Fulk A. Romero-Alvarez D. Saymeh Q. A. Saint Onge J. Peterson A. T. Agusto F. B. (2021). Using Google Health Trends to investigate COVID19 incidence in Africa. medRxiv. [preprint]. 10.1101/2021.03.26.21254369 35671301 PMC9173636

[B10] Google Trends. (2018). How Data Is Adjusted. Available online at: https://support.google.com/trends/answer/4365533?hl=en (accessed May 22, 2018).

[B11] Hyndman R. J. Athanasopoulos G. (2018). Forecasting: Principles and Practice. Melbourne, VIC: OTexts.

[B12] Hyndman R. J. Khandakar Y. (2008). Automatic time series forecasting: the forecast package for R. J. Stat. Softw. 27, 1–22. 10.18637/jss.v027.i03

[B13] Johansson M. A. Reich N. G. Hota A. Brownstein J. S. Santillana M. (2016). Evaluating the performance of infectious disease forecasts: a comparison of climate-driven and seasonal dengue forecasts for Mexico. Sci. Rep. 6, 1–11. 10.1038/srep33707 27665707 PMC5036038

[B14] Kandula S. Shaman J. (2019). Near-term forecasts of influenza-like illness: an evaluation of autoregressive time series approaches. Epidemics 27, 41–51. 10.1016/j.epidem.2019.01.002 30792135

[B15] Kane M. J. Price N. Scotch M. Rabinowitz P. (2014). Comparison of ARIMA and Random Forest time series models for prediction of avian influenza H5N1 outbreaks. BMC Bioinformatics 15, 1–9. 10.1186/1471-2105-15-276 25123979 PMC4152592

[B16] Kwiatkowski D. Phillips P. C. Schmidt P. Shin Y. (1992). Testing the null hypothesis of stationarity against the alternative of a unit root: How sure are we that economic time series have a unit root? J. Econom. 54, 159–178. 10.1016/0304-4076(92)90104-Y

[B17] Lu F. S. Hou S. Baltrusaitis K. Shah M. Leskovec J. Hawkins J.. (2018). Accurate influenza monitoring and forecasting using novel internet data streams: a case study in the Boston Metropolis. JMIR Public Health Surveill. 4, e8950. 10.2196/publichealth.8950 29317382 PMC5780615

[B18] Mavragani A. Gkillas K. (2020). COVID-19 predictability in the United States using Google Trends time series. Sci. Rep. 10, 1–12. 10.1038/s41598-020-77275-9 33244028 PMC7692493

[B19] Mavragani A. Ochoa G. (2018a). Forecasting AIDS prevalence in the United States using online search traffic data. J. Big Data 5, 1–21. 10.1186/s40537-018-0126-7

[B20] Mavragani A. Ochoa G. (2018b). Infoveillance of infectious diseases in USA: STDs, tuberculosis, and hepatitis. J. Big Data 5, 1–23. 10.1186/s40537-018-0140-9

[B21] Mavragani A. Ochoa G. Tsagarakis K. P. (2018a). Assessing the methods, tools, and statistical approaches in Google Trends research: systematic review. J. Med. Internet Res. 20, e9366. 10.2196/jmir.9366 30401664 PMC6246971

[B22] Mavragani A. Sampri A. Sypsa K. Tsagarakis K. P. (2018b). Integrating smart health in the us health care system: infodemiology study of asthma monitoring in the google era. JMIR Public Health Surveill. 4, e8726. 10.2196/publichealth.8726 29530839 PMC5869181

[B23] Midekisa A. Senay G. Henebry G. M. Semuniguse P. Wimberly M. C. (2012). Remote sensing-based time series models for malaria early warning in the highlands of Ethiopia. Malar. J. 11, 1–10. 10.1186/1475-2875-11-165 22583705 PMC3493314

[B24] Nann D. Walker M. Frauenfeld L. Ferenci T. Sulyok M. (2021). Forecasting the future number of pertussis cases using data from Google Trends. Heliyon 7, e08386. 10.1016/j.heliyon.2021.e08386 34825092 PMC8605298

[B25] NCDC (2020). COVID-19 Outbreak in Nigeria: Situation Reports. Available online at: https://ncdc.gov.ng/diseases/sitreps (accessed February 28, 2022).

[B26] Nuti S. V. Wayda B. Ranasinghe I. Wang S. Dreyer R. P. Chen S. I.. (2014). The use of google trends in health care research: a systematic review. PLoS ONE 9, e109583. 10.1371/journal.pone.0109583 25337815 PMC4215636

[B27] Pan Z. Nguyen H. L. Abu-Gellban H. Zhang Y. (2020). “Google trends analysis of covid-19 pandemic,” in 2020 IEEE International Conference on Big Data (Big Data). IEEE, 3438–3446. 10.1109/BigData50022.2020.937785227295638

[B28] R Core Team (2020). R: A Language and Environment for Statistical Computing. Vienna, Austria: R Foundation for Statistical Computing.

[B29] Ren H. Li J. Yuan Z.-A. Hu J.-Y. Yu Y. Lu Y.-H. (2013). The development of a combined mathematical model to forecast the incidence of hepatitis E in Shanghai, China. BMC Infect. Dis. 13, 1–6. 10.1186/1471-2334-13-421 24010871 PMC3847129

[B30] Rovetta A. Castaldo L. (2020). The impact of COVID-19 on Italian web users: a quantitative analysis of regional hygiene interest and emotional response. Cureus 12, e10719. 10.7759/cureus.10719 33150116 PMC7605457

[B31] Salathé M. (2018). Digital epidemiology: what is it, and where is it going? Life Sci. Soc. Policy 14, 1–5. 10.1186/s40504-017-0065-7 29302758 PMC5754279

[B32] Satpathy P. Kumar S. Prasad P. (2021). Suitability of Google Trends™ for digital surveillance during ongoing COVID-19 epidemic: a case study from India. Disaster Med. Public Health Prep. 1–10. 10.1017/dmp.2021.249 34343467 PMC8460424

[B33] Song X. Xiao J. Deng J. Kang Q. Zhang Y. Xu J. (2016). Time series analysis of influenza incidence in Chinese provinces from 2004 to 2011. Medicine 95, e3929. 10.1097/MD.0000000000003929 27367989 PMC4937903

[B34] Sulyok M. Richter H. Sulyok Z. Kapitány-Fövény M. Walker M. D. (2020). Predicting tick-borne encephalitis using Google Trends. Ticks Tick Borne Dis. 11, 101306. 10.1016/j.ttbdis.2019.101306 31624027

[B35] Teng Y. Bi D. Xie G. Jin Y. Huang Y. Lin B.. (2017). Dynamic forecasting of Zika epidemics using Google Trends. PLoS ONE 12, e0165085. 10.1371/journal.pone.0165085 28060809 PMC5217860

[B36] Van Lent L. G. Sungur H. Kunneman F. A. Van De Velde B. Das E. (2017). Too far to care? Measuring public attention and fear for Ebola using Twitter. J. Med. Internet Res. 19, e7219. 10.2196/jmir.7219 28611015 PMC5487741

[B37] Wongkoblap A. Vadillo M. A. Curcin V. (2017). Researching mental health disorders in the era of social media: systematic review. J. Med. Internet Res. 19, e228. 10.2196/jmir.7215 28663166 PMC5509952

[B38] Wongkoon S. Jaroensutasinee M. Jaroensutasinee K. (2012). Assessing the temporal modelling for prediction of dengue infection in northern and northeastern, Thailand. Trop. Biomed. 29, 339–348. 23018496

[B39] Worldometer (2022). Coronavirus Update (Live): COVID-19 Virus Outbreak. Available online at: https://www.worldometers.info/coronavirus/country/nigeria (accessed February 27, 2022).

[B40] Xu C. Yang H. Sun L. Cao X. Hou Y. Cai Q.. (2020). Detecting lung cancer trends by leveraging real-world and internet-based data: Infodemiology study. J. Med. Internet Res. 22, e16184. 10.2196/16184 32163035 PMC7099398

[B41] Zhang Y. Bambrick H. Mengersen K. Tong S. Hu W. (2018). Using Google Trends and ambient temperature to predict seasonal influenza outbreaks. Environ. Int. 117, 284–291. 10.1016/j.envint.2018.05.016 29778013

