# Supplementary material for: Modeling COVID-19 incidence with Google Trends
Source: Front Res Metr Anal. 2022 Sep 15;7:1003972. doi: 10.3389/frma.2022.1003972 (PMC9520600; doi:10.3389/frma.2022.1003972)

KPSS Test for Level Stationarity

data: difftsdat

KPSS Level = 0.14254, Truncation lag parameter = 3, p-value = 0.1

KPSS Test for Trend Stationarity

data: difftsdat

KPSS Trend = 0.099758, Truncation lag parameter = 3, p-value = 0.1

Ljung-Box test

data: Residuals from ARIMA(2,1,0)

Q* = 10.878, df = 8, p-value = 0.2087

Model df: 2. Total lags used: 10


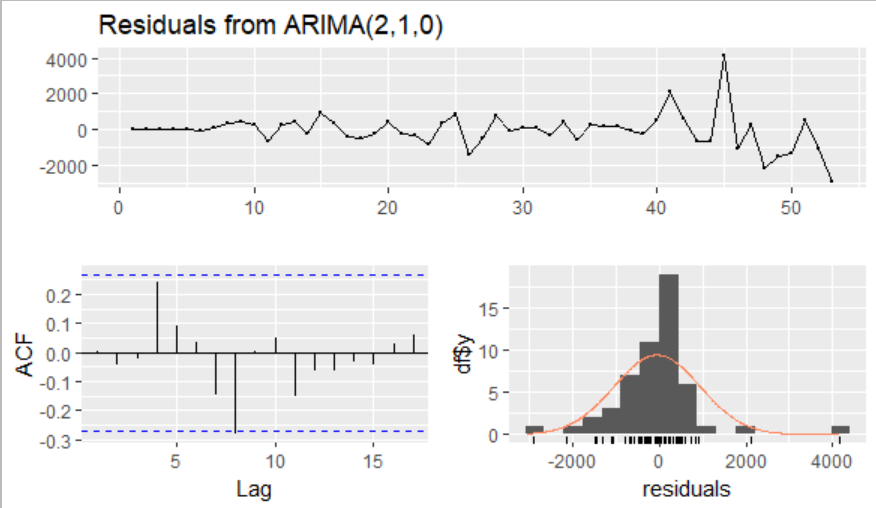

Supplement: Supplementary file 1 [file Table_1.DOCX]
